# Supplementary material for: Venomix: a simple bioinformatic pipeline for identifying and characterizing toxin gene candidates from transcriptomic data
Source: PeerJ. 2018 Jul 31;6:e5361. doi: 10.7717/peerj.5361 (PMC6074769; doi:10.7717/peerj.5361)
Supplement: Supplemental Information 6 [file peerj-06-5361-s006.gz › FinalOutput_1E-6/Venom_prothrombin_activator_pseutarin-C_catalytic_subunit_12/finaltree.pdf]

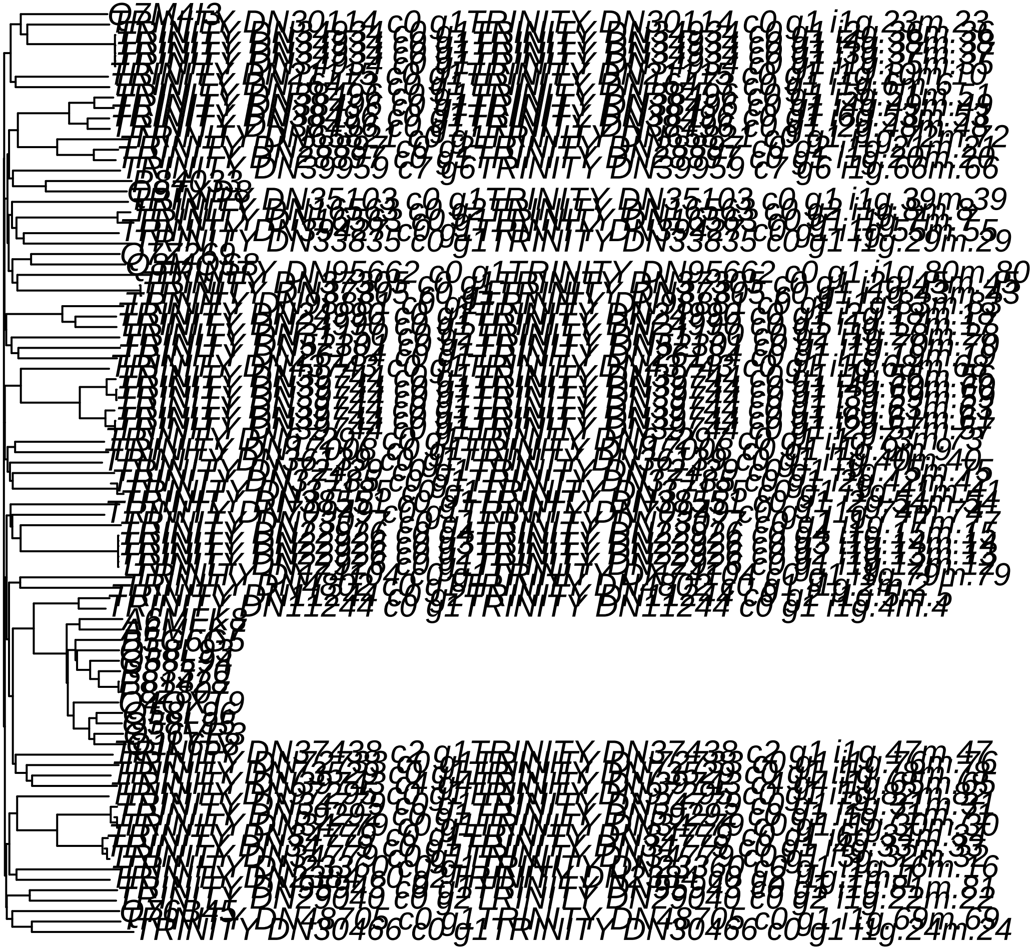
Q7M4I3
TRINITY DN30114 c0 g1TRINITY DN30114 c0 g1 i1g.23m.23
TRINITY DN39959 c7 g6TRINITY DN39959 c7 g6 i1g.66m.66
TRINITY DN35103 c0 g1TRINITY DN35103 c0 g1 i1g.39m.39
TRINITY DN33835 c0 g1TRINITY DN33835 c0 g1 i1g.29m.29
TRINITY DN95662 c0 g1TRINITY DN95662 c0 g1 i1g.80m.80
TRINITY DN11244 c0 g1TRINITY DN11244 c0 g1 i1g.4m.4
TRINITY DN37438 c2 g1TRINITY DN37438 c2 g1 i1g.47m.47
TRINITY DN29040 c0 g2TRINITY DN29040 c0 g2 i1g.22m.22
TRINITY DN48705 c0 g1TRINITY DN48705 c0 g1 i1g.69m.69
TRINITY DN30466 c0 g1TRINITY DN30466 c0 g1 i1g.24m.24
